# Enhanced room temperature ferromagnetism and green photoluminescence in Cu doped ZnO thin film synthesised by neutral beam sputtering

**DOI:** 10.1038/s41598-019-43184-9

**Published:** 2019-04-30

**Authors:** D. C. Agarwal, U. B. Singh, Srashti Gupta, Rahul Singhal, P. K. Kulriya, Fouran Singh, A. Tripathi, Jitendra Singh, U. S. Joshi, D. K. Avasthi

**Affiliations:** 1 Department of Physics, Sant Longowal Institute of Engineering & Technology, Longowal, Sangrur, Punjab 148106 India; 2 Material Science, Inter-University Accelerator Centre, New Delhi, 110 067 India; 3 Department of Physics, Deen Dayal Upadhyaya Gorakhpur University, Gorakhpur, U.P. 273009 India; 4 Deptartment of Physics & Astrophysics, University of Delhi, Delhi, 110007 India; 5 Department of Physics, Malaviya National Institute of Technology, Jaipur, Rajasthan 302017 India; 6 CSIR-Central Electronics Engineering Research Institute, Pilani, Rajasthan 333031 India; 7 Department of Physics, Gujarat University, Ahmedabad, 380009 India; 8 Amity Institute of Nanotechnology, Amity University, Noida, Uttar Pradesh 201303 India

**Keywords:** Nanoscience and technology, Materials science

## Abstract

The Cu (3 to 15 at%) is incorporated into ZnO thin film by atomic beam co-sputtering has been investigated for enhancement in room temperature ferromagnetism and green photo-luminance. These Cu-ZnO thin films examined with Raman spectroscopy, X-Ray Diffraction (XRD), UV-Visible spectroscopy, Hall measurement, magnetic force microscopy (MFM) and magnetic hysteresis. Raman spectroscopy, XRD confirms wurtzite structure and improvement in the crystallinity of ZnO upto 7% Cu. Further increase in Cu concentration results in growth in Cu nanoparticles. On increasing Cu concentration, there is decrement in transparency and increase in band gap with increase in n-type carrier concentration as confirmed from UV-Visible and Hall measurement studies. Magnetic measurement exhibited unique feature of room temperature ferromagnetic ordering in undoped and doped sample upto 3% Cu. The enhancement in magnetic moment as well as green emission in photoluminescence response with increase in Cu doping indicates that generation of large defects in ZnO by Cu doping, which can be attributed to combined effect of the presence of oxygen vacancies and/or structural inhomogeneity as well as formation of bound magnetic polarons. Importantly, synthesised Cu doped ZnO thin films can be used as spin LEDs and switchable spin-laser diodes.

## Introduction

Metal oxides give an opportunity to extend the performance of today’s semiconductor devices which are widely used in high frequency, high power and high temperature applications. One can also visualize the concept of novel electronic or optoelectronic devices which are based on hetero junctions and/or combinations with today’s technology. Since last decades there was a restriction to access the high-quality wide band gap material but recent progress in the growth of wide band gap semiconductor such as zinc oxide (ZnO), titanium oxide (TiO_2_) and tin oxide (SnO_2_) etc., made these materials available for device applications. ZnO is a wide band gap semiconductor which is widely studied material due to its unique properties with high breakdown strength and high saturation velocity, which make it more attractive for technological purpose^1–5^.

ZnO based room temperature and low temperature optical UV lasing has already been realized and demonstrated^6^, although development of efficient electrical lasing is restricted due to the realization of good, *p*-type conduction in ZnO material. Furthermore, radiation hardness of ZnO is better than other common semiconductor materials, such as Si, GaAs, CdS, and even GaN; thus, it is more suitable for space applications. Like many other transparent metal oxides, inclusion of metal nanoparticles in ZnO offers attractive features of optical responses including nonlinear effects linked to absorption by Surface Plasmon Resonance. Doping of various impurity elements in ZnO shows different functions and improved properties of semiconductor compounds such as electrical, optical and mechanical, which facilitate the development of many electronic and optoelectronic devices^7–9^. It is expected that doping can enhance the diversity of applications of these semiconductors. Impurity doped ZnO is promising because this is composed of relatively abundant and ecologically friendly elements compared to other binary compounds. Cu is an attractive dopant due to its high electronic conductivity, being relatively cheaper and abundant on the Earth’s crust. Doping of Cu also helps in achieving p-type ZnO. Cu atoms, act as deep acceptor due to its substitution for the Zn atom and energy level of this acceptor (Cu^2+^_Zn_) located at 0.17 eV below the bottom of the conduction band^10^ and also above the valance band, an acceptor level with energy 0.45 eV is formed^11^. Photoluminescence properties of ZnO can be tailored by doping of foreign elements in ZnO^12–14^ and green emission, is reported on Cu doping in ZnO, however its mechanism is the part of focused discussion^15,16^. On the other hand, metal doped ZnO is still led to intense research interest of researchers due to its application in spintronic devices. Spintronic application requires the intrinsic ferromagnetic property rather than due to the presence of metal dopants. There is always a debate in scientific community for the use of transition metal ions (Co, Fe, Ni, Mn) as dopants in ZnO because it become difficult to analyze the occurrence of ferromagnetic nature due to the intrinsic property or due to the transition metal ions^17–20^. In past, evidence of intrinsic ferromagnetism arises in individual ZnO nanoparticles when doped with Co while in addition Fe doping can not show any intrinsic ferromagnetism^21^. To overcome these issues, there is requirement of such dopant which does not show ferromagnetism. Cu-doped ZnO (ZnO:Cu) does not have ferromagnetic impurities due to the non metallic nature of Cu as well as its oxides are non ferromagnetic leading to form an intrinsic dilute magnetic semiconductor^22,23^. Mutual ferromagnetic-ferroelectric coupling is recently reported in Cu-doped ZnO^24^. Robust room temperature ferromagnetism (RTFM) in Cu doped ZnO can lead to the fabrication of spin LEDs and switchable spin-laser diodes. On the other hand, there are many theoretical models to explain ferromagnetism in dilute magnetic semiconductors. RKKY model^25^ which explains magnetic interaction between single localized magnetic ion and delocalized conduction band electrons. Mean field Zener theory^26^ which is based on Zener model and RKKY model in which delocalized hole carriers mediate a RKKY like interaction among localized transition metal ion resulting in ferromagnetism. Mechanism of double exchange model is given by Zener^27^ based on the hopping of electrons between two neighbor transition metal ions i.e. describes a system of localized spins interacting with spins of conducting electrons, to explain the strong correlation between movement of charge and spin polarization of the magnetic lattice ions. Indirect double exchange model^28^ which was based on first principle calculations proposed that alignment of localized large moments of Cu in the vicinity of Vo are mediated by large sized vacancy orbitals. Bound magnetic polarons (BMP) model^29–31^ is used to explain ferromagnetic ordering in transition metal doped ZnO, in this polarons is a collections of electrons or holes which are bound to impurity atoms through exchange interactions with in an orbit.

However, tireless efforts are going on by several research groups on room temperature ferromagnetism and green luminescence in Cu doped ZnO even then their origin remain divisive among the researchers. Moreover, Keavney *et al*. explained RTFM in Cu doped ZnO thin films on the basis of spin polarization on the Cu 3d and O 2p states^32^. Such intriguing and controversial literature on green luminescence and RTFM in Cu doped ZnO suggest that Cu-doped ZnO system requires more detailed study and warrants further investigations. Ferromagnetic semiconductor operative at room temperature are needed for practical realization of spintronic devices. In spite of large number of literature ZnO based DMS systems there is no congruent opinion on the origin of ferromagnetism in the systems synthesized by different techniques and researchers. Contentious outcome of different experimental results recommends that the properties of DMS systems are very sensitive to synthesis technique and process parameters. The hunt of material for spintronic device is still breathing. The knowledge of the optical properties of Cu doped ZnO thin film with RTFM is enormously vital and demands in depth study for the design and analysis of various optical and optoelectronic devices.

The present work focuses on the structural, optical, electrical and magnetic properties of ZnO:Cu films synthesized using atom beam co-sputtering^33,34^ by varying Cu atomic fraction. The efforts have been made to correlate all the physical properties of Cu doped ZnO and the basic mechanism for observation of RTFM, and green luminescence in ZnO thin films is explained comprehensively.

## Results and Discussions

**Figure 1:**
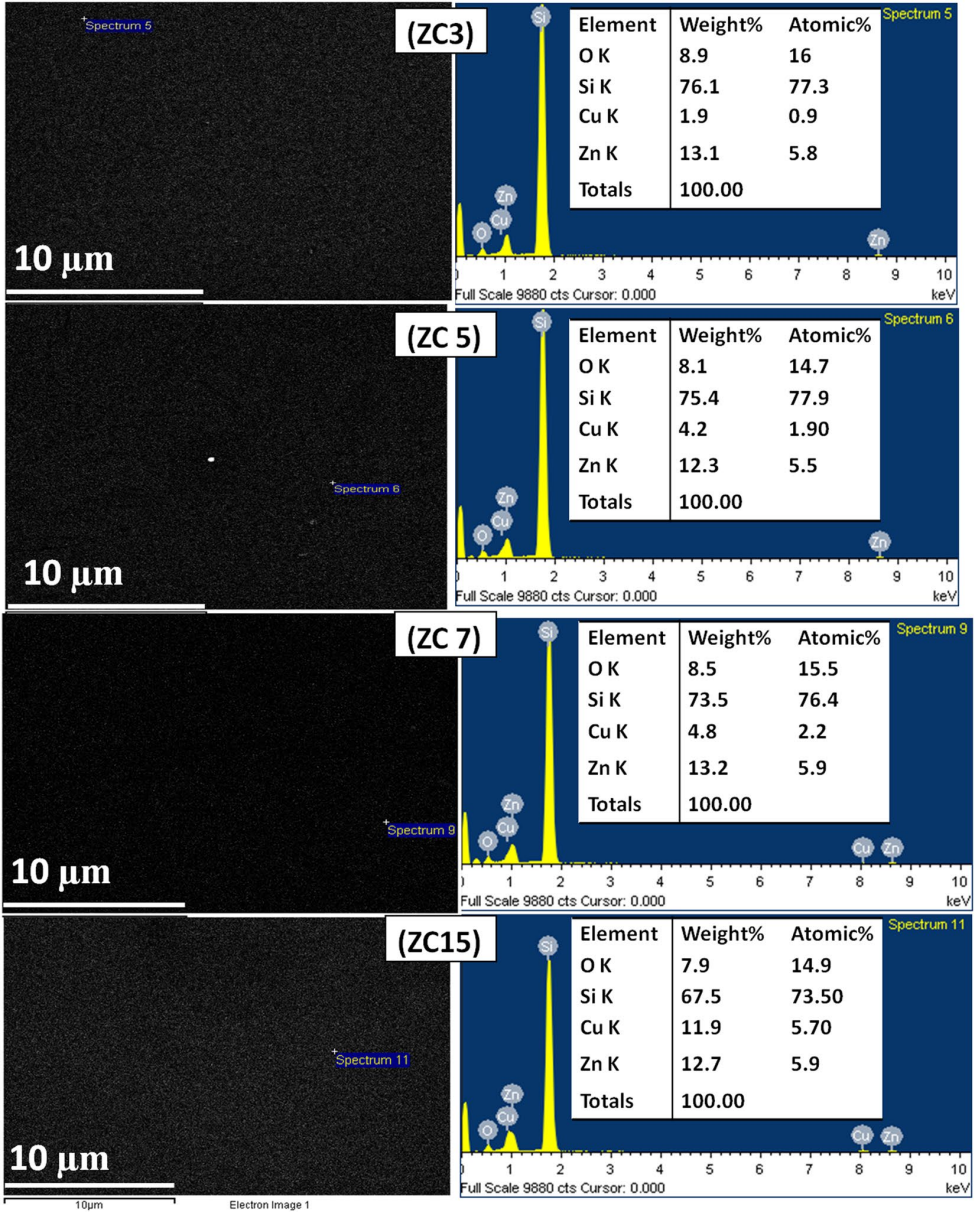
SEM EDX of all samples [(**a**) ZC3, (**b**) ZC5, (**c**) ZC7 and (**d**) ZC15] shows SEM images (left), EDX (right) with elemental composition (O, Cu, Si, Zn) without any ferromagnetic impurity, uniform distribution of Cu in ZnO with minor variation ± 0.1%.

The composition of the Cu doped ZnO thin films is determined using SEM EDX of all samples (ZC3, ZC5, ZC7, ZC15) as shown in Fig. 1. The EDX analysis shows presence of only Zn, O, Cu and Si. There are no detectable traces of any ferromagnetic impurity in the ZnO: Cu nanocomposites. The Si signal appeared from the substrate. SEM EDX has been done at different places and the composition of Zn and Cu obtained were same on the entire surface of the sample with minor variation ~ ± 0.1%, indicating the uniform distribution of Cu dopant in the nanocomposite films. However, quantification of O is difficult, as oxygen is also present in SiO_2_ layer over Si substrate due to the exposure of Si substrate in the air during transportation. The EDX result also shows variation in Zn/O ratio and Cu/Zn ratio with variation in doping concentration of Cu. Quantitative elemental analysis of ZnO:Cu nanocomposite films are given in Table 1. It confirms that only Zn, O and Cu are present in the film and their concentration is not as per stoichiometric proportion. The ratio of Zn/O is found to increase, whereas ratio of Zn/Cu is decreased with increase in Cu concentration. The increase in Zn/O ratio suggests that oxygen deficiency increases with an increase in Cu concentration in the film. The deficiency or excess of the constituent material results in distorted band structure which could be responsible for the enhancement in the conductivity of the film.

**Table 1:** Carrier density, carrier mobility, and resistivity from Hall effect measurement and elemental composition of Zn, Cu, O determined by EDX measurement of ZnO and Cu doped ZnO thin films.

Films	Carrier density (n_d_) cm^−3^	Carrier Mobility (μ) cm^2^v^−1^s^−1^	Resistivity (Ω.cm)	Zn		O		Cu	
				at%	wt%	at%	wt%	at%	wt%
ZnO	6.0 * 10^17^	4.5	1.2 * 10^−1^	3.2	7.5	9.30	5.3	…..	…..
ZC3	2.6 * 10^17^	5.3	4.6	5.8	13.1	16.0	8.9	0.9	1.9
ZC5	1.4 * 10^19^	56.9	7.6 * 10^−3^	5.5	12.3	14.7	8.1	1.9	4.2
ZC7	4.5 * 10^19^	32.0	4.4 * 10^−3^	5.9	13.2	15.5	8.5	2.2	4.8
ZC15				5.9	12.7	14.9	7.9	5.7	11.9

**Figure 2:**
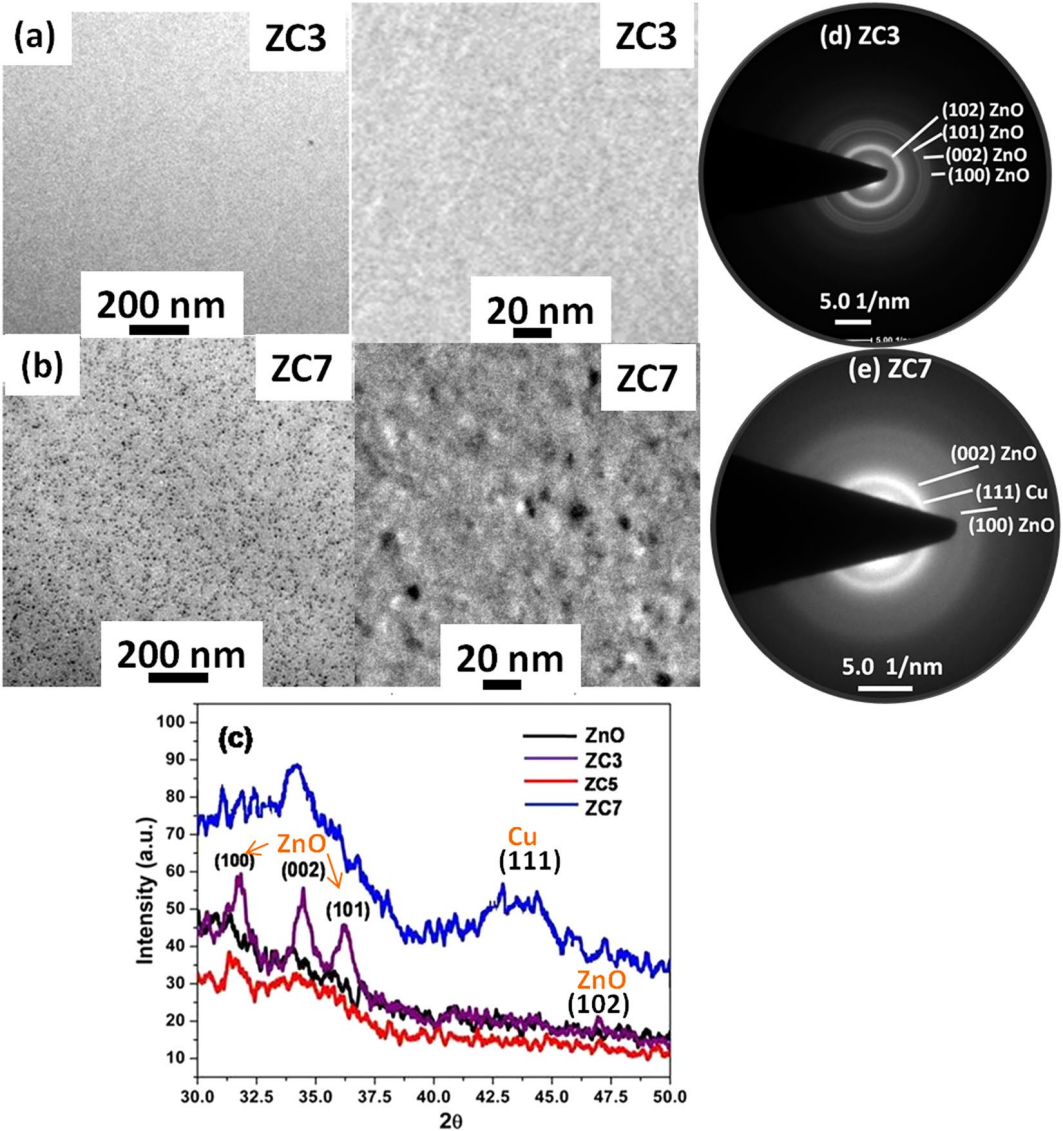
TEM images [scale 200 nm (left) and 20 nm (right)] of Cu: ZnO thin film shows (**a**) untraceable Cu nano particle in ZC3 sample, (**b**) Cu nanoparticles (<10 nm) in ZC7 sample, (**c**) X-ray diffraction pattern of ZnO:Cu sample showing the crystalline nature of ZnO in low Cu concentration (≤5%) with no reflection of Cu and its oxide phases, and for high Cu concentration (≥7%) decrement of crystallinity of ZnO with broad reflection (at 2θ~43.4) of metallic Cu nano particle. TEM SAED pattern supports the XRD outcomes, (**d**) ZC3 sample represent the rings of crystalline ZnO, (**e**) ZC7 sample represent the ring of Cu alongwith ZnO.

Structural properties of un-doped ZnO thin film and the effect post deposition treatment like thermal and swift heavy ion irradiation have been reported elsewhere^33^. Figure 2(a,b) show a plan-view bright field TEM image (with 200 nm and 20 nm scale ruler) of the ZC3 and ZC7 samples deposited on carbon coated grids, respectively. The absence of nanoparticles in ZC3 sample indicates that Cu is either at the substitutional site of the ZnO lattice or it is in the form of atoms and small clusters with sizes below the detection limit. The formation of nanoparticles in ZC7 sample can be clearly seen in TEM micrograph (Fig. 2(b)). The XRD patterns of the ZnO and ZnO:Cu systems with different Cu concentration are shown in Fig. 2(c). The crystallinity of nanocrystalline ZnO film is gradually improved with increase in Cu concentration. For lowest concentration of Cu, diffraction peaks appearing at the peak position of 31.7°, 34.4° and 36.2° correspond to (100), (002) and (101) crystal planes of ZnO in wurtzite structure. XRD of ZC7 sample shows hump at 2θ = 43.4° corresponds to Cu (111) plane, which is not visible in other samples. Broadening of the (111) plane may be due to the formation of Cu nanoparticle in ZC7 sample. XRD data is also supported by TEM and SAED pattern. SAED pattern of ZC3 (Fig. 2(d)) and ZC7 (Fig. 2(e)) are shown. The appearance of sharp rings with little diffusion in ZC3 reveals the crystalline nature. A hcp structure of ZnO is proclaimed by the presence of (100), (002), (101), (102)…..diffraction rings after the indexation. No rings correspond to Cu and its oxide phases are detected in ZC3 sample. Furthermore, SAED pattern of ZC7 contain more diffuse rings, which can be assigned to an amorphous or nanocrystalline structure with very small grain size. Two rings are attributed to (002), (100) planes of hcp ZnO and other one is indexed to (111) plane of fcc Cu.

It is well known that ionic radii of Cu^+2^ (0.057 nm) is smaller than the Zn^+2^ (0.06 nm) and hence substitution of Cu at the Zn site results in increase in lattice constant. It also helps in the reduction of the strain induced due to large lattice mismatch between ZnO and Si substrate, which is already present in the sputtered ZnO film, which arises due to large lattice mismatch between ZnO and Si substrate. Here, ZnO is grown as non-epitaxial layers due to discontinuous layers formed by the addition of Cu nanoclusters. Hence there is a change in stress which leads to the decrease in lattice constant due to the liberation of the stress in ZnO film. XRD of ZnO:Cu samples shows that within the detection limit of XRD, no other peaks corresponding to copper and its related secondary or impurity phase were found, which reveals that the substitution of Cu does not affect the wurtzite structure of zinc oxide at lowest Cu concentration. Typically, with the increase of Cu concentration beyond 3%, the lattice of ZnO is distorted as some of the reflections of ZnO disappeared and (002) reflection became broad. The formation Cu nanoparticles are also evidenced from the reflection of Cu in the XRD spectrum of the sample having more than 7% Cu concentration.

**Figure 3:**
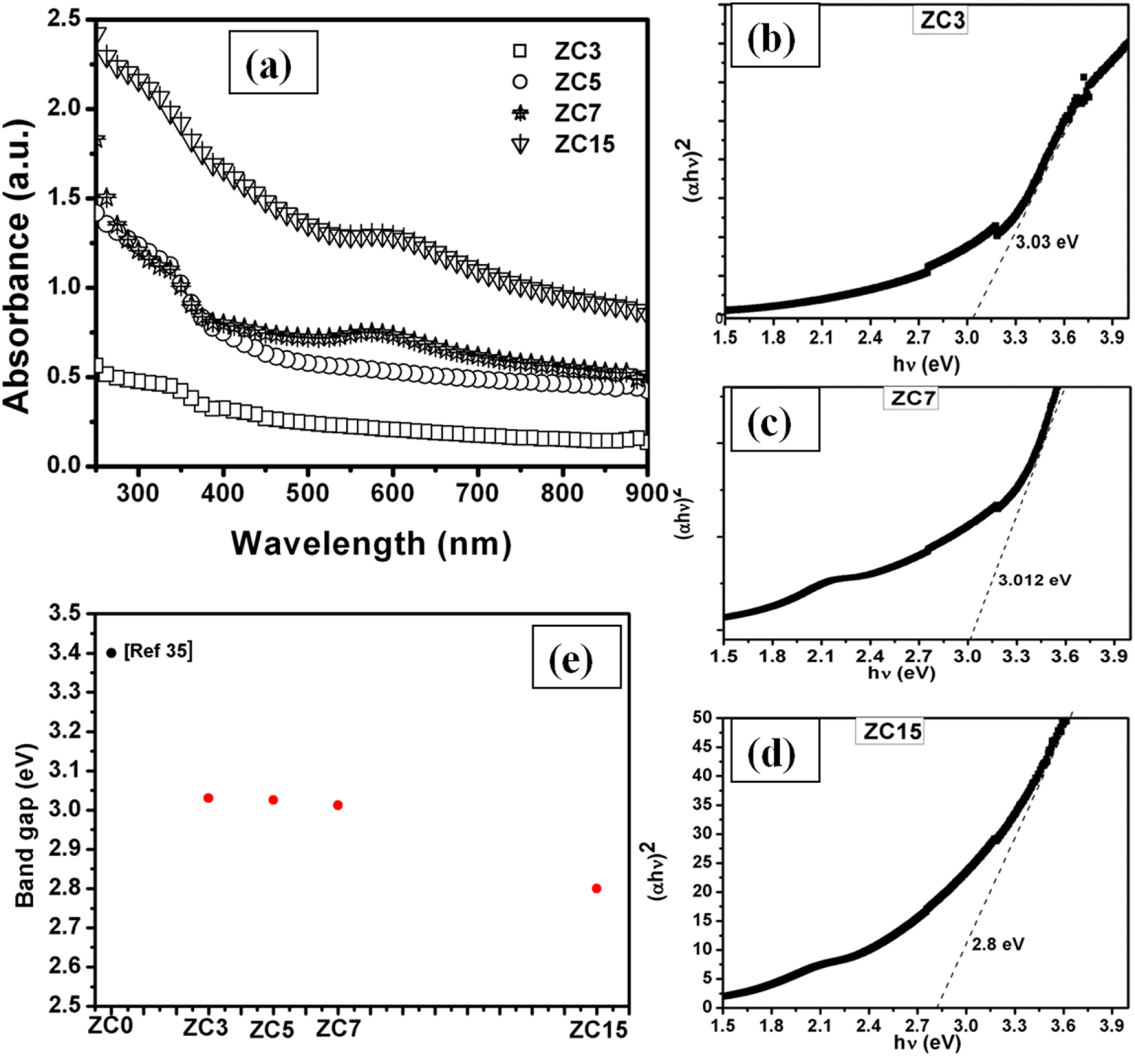
(**a**) Absorption spectra of Cu doped ZnO thin film shows the band edge of ZnO in all samples alongwith SPR at higher Cu concentration (≥7%) and tauc’s plot with optical band gap energy of (**b**) ZC3, (**c**) ZC7, (**d**) ZC15 sample is shown, (**e**) determination of band gap from tauc’s plot shows the decrease in band gap with increase in Cu concentration.

All optical absorption spectra of ZnO:Cu films for varying Cu concentration examined at room temperature have sharp band edge in UV region as indicated by the Fig. 3(a). Observed absorption band edge has been changed from 405 nm to 445 nm as Cu concentration varies from 3% to 15%. The direct band gap of the sample was determined using the Tauc’s plot from the equation:

1 $${\rm{\alpha }}{\rm{h}}{\rm{\upsilon }}={\rm{A}}{({\rm{h}}{\rm{\upsilon }}-{{\rm{E}}}_{{\rm{g}}})}^{{\rm{r}}}$$

Where, α is absorption coefficient, A is constant, r is ½, E_g_ is band gap, h is plank constant, υ is the photon frequency.

Tauc’s plot has been used to determine the optical energy band gap of pure ZnO and Cu doped ZnO as shown in Fig. 3(b to d). The band gap of atom beam sputtered ZnO thin film is ~3.4 eV which is reported in our previous work^35^. Band gap is little bit higher than the bulk ZnO that may be due to the quantum confinement in nanocrystalline ZnO. In case of Cu doping, the value of band gap decreases with increase in the Cu concentration in ZnO (band gap of ZC3-3.03 eV, ZC5-3.026 eV, ZC7-3.01 eV, ZC15-2.8 eV) as shown in Fig. 3(e). In earlier reports, similar mitigation of the band gap with increasing Cu concentration has also been reported^36,37^.

It is clearly seen that the incorporation of Cu in ZnO reduces the transparency. The absorption is increased in visible region with increase in Cu content in the film. This visible absorption may be attributed to the intra-band transition of Cu 3d band and Zn 4 s states in the conduction band. The reduction in transmittance may also be attributed to the shallower nature of Cu 3d orbital than Zn 3d orbital^38^. When Cu atom takes the Zn site in ZnO, it causes two possible effects: (a) narrowing the fundamental band gap of the system due to the strong d–p coupling between Cu and O, which in turn leads O 2p level up and (b) an impurity band above the valence band of ZnO is formed by the Cu 3d orbital. These two effects are clearly seen for heavily doped ZnO:Cu thin films. The absorbance spectra contains two parts—one originated from the direct fundamental band gap and the other originated from mixed impurity states between band gap, which increase the absorption of the system in visible region. Therefore, Cu doping enhances the photo catalytic activity of ZnO due to the intensive and wide wavelength visible light absorption, which, in turn, would also make ZnO a potential candidate for photo electrochemical application^39^. Thus, UV measurement was performed to show the change in band gap of ZnO by Cu doping and also study the different phases of Cu like, Cu nanoparticle which is confirmed by SPR (characteristic feature of nano particle) and oxide phases (CuO and Cu_2_O) which can be ruled out due to the invisibility of any band edge corresponding to oxide phase of Cu. In transition metal doped ZnO samples, there is change in sp-d exchange interaction between the band electrons and the localized d-electron of the Cu^2+^ ions which leads to the red shift in band edge^40^. For small volume fraction of Cu up to 5%, the sample exhibits the characteristics of ZnO with a red shift and tailing in the band edge absorption. However, the ZnO-Cu nanocomposites with higher concentration exhibit both the semiconductor and metallic behavior with temperature. The optical absorption spectra indicate the presence of a well-defined ZnO band gap absorption feature along with the feature of surface plasmon resonance (SPR) of Cu, which confirm the formation of Cu nanoparticles. The SPR peak shows a red shift from 580 nm to 592 nm on increasing the Cu concentration from 7% to 15%. It can be explained as for higher Cu content there is an accumulation of access electrons on the ZnO/Cu particles which leads to the equalization of collective Fermi level of semiconductor/metal system and the potential of conduction band of semiconductor^41^. Thus, shift in SPR peak in ZnO/Cu system for higher Cu content samples is due the accumulation of access electron on ZnO/Cu which results in increase in potentials of conduction band of semiconductor and metal.

**Figure 4:**
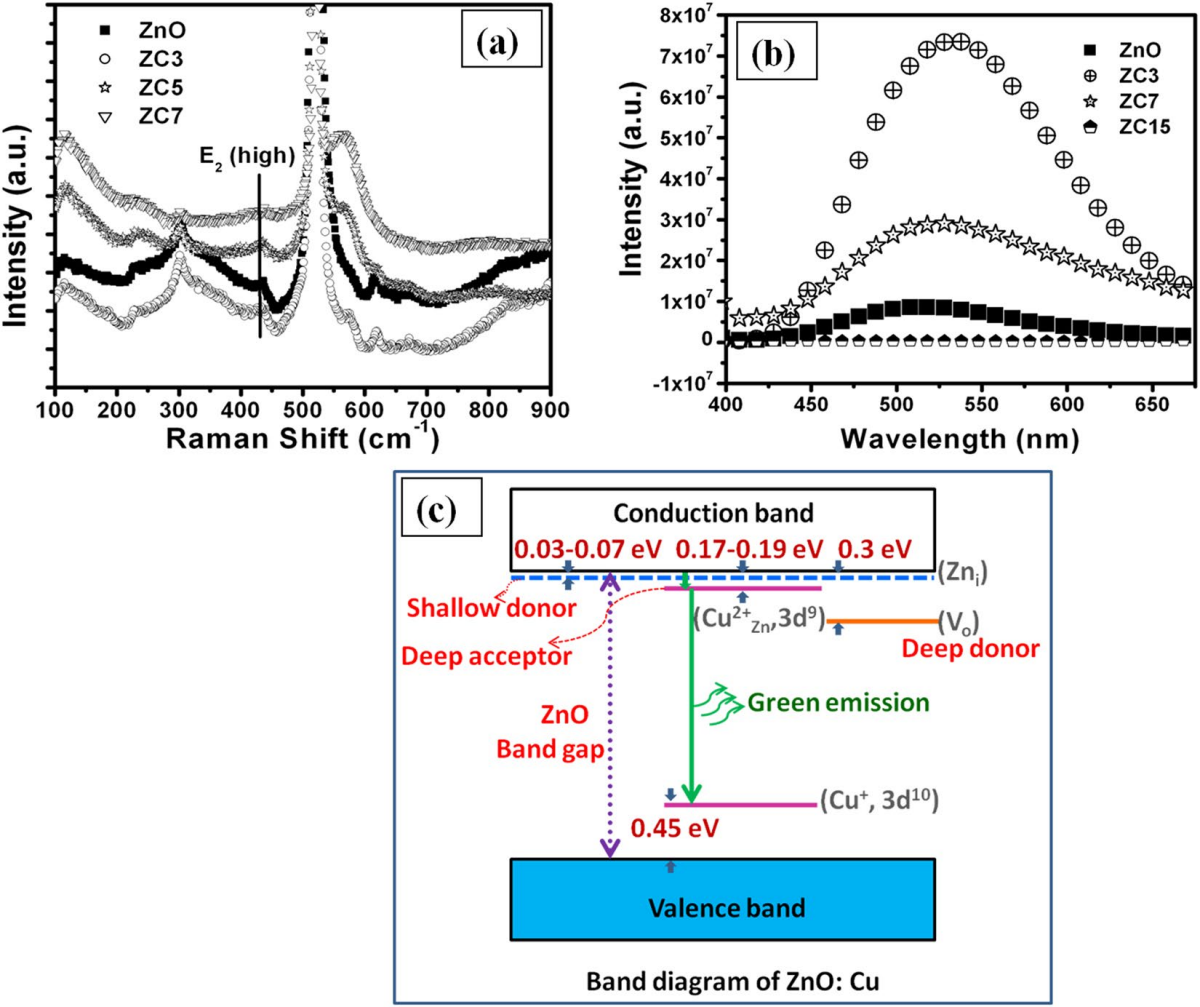
(**a**) Raman spectra of ZnO:Cu thin film shows E_2_ (high) phonon mode at 435 cm^−1^ which is weak, broad and slightly shifted to lower frequency, indicating nanocrystalline nature of ZnO, (**b**) Photoluminescence spectra of broad green band with high intensity at lowest Cu concentration, (**c**) Schematic of energy level diagram of Cu doped ZnO system.

The non-resonant Raman spectra of ZnO:Cu nanocomposite films deposited on Si substrate are examined and shown in Fig. 4(a). The wurtzite ZnO belongs to the space group of C_6v_, with six active Raman modes of E_2L_ + E_2H_ + A_1T_ + A_1L_ + E_1T_ + E_1L_^42^. The peak appearing at wave number of 435 cm^−1^ correspond to E_2_(high) phonon mode, typical for the hexagonal phase of the ZnO. This mode is very weak and broad and also slightly shifted to lower frequency, which indicates nanocrystalline nature of ZnO. The E_2_(high) phonon mode is disappeared at high concentration of Cu (~15 at. %). Alongwith the characteristic mode of ZnO, a few second order modes are also observed. The modes at 233 and 300 cm^−1^ are related to B_1_ (high) −B_2_ (low) phonon mode frequencies^43^. A very broad and intensive signal at 573 cm^−1^ is also observed in contrast to the pure ZnO^33^, which is assigned to A_1_(LO) mode. This mode generally originates due to the defects such as oxygen vacancies in the ZnO. Singh *et. al*.^44^ also showed that A_1_(LO) originates due to incorporation of huge disorder and defects after the application of swift heavy ion irradiation of ZnO. This peak becomes more intense and broad with increase in Cu concentration, indicating the increase in concentration of oxygen vacancies. The Raman results are in good agreement with XRD, which shows distortion of ZnO lattice and creation of defects at high Cu concentration. Also similar to XRD results, there is no signal corresponding to copper oxide or other copper compounds in the Raman spectra. Since, the light penetration depth is larger than the thickness of the ZnO films, a pronounced mode E_2_ (high) of the Si substrate is also observed at the wave number of 521 cm^−1^.

ZnO is a traditional n-type semiconductor with the electrons to move in the conduction band as a charge carrier due to native defects like oxygen vacancies and zinc interstitial, which are acting as a shallow donor and electron supplier. Cu doping is believed to make p-type ZnO. In order to study the electrical properties of ZnO:Cu nanocomposites grown on quartz substrate, Hall measurements were performed in a Van der Pauw four-point configuration at room temperature and the corresponding results are listed in Table 1. It may be noticed that all the films exhibit n-type conductivity. The conductivity of ZnO film is decreased by one order of magnitude and carrier concentration and mobility is also reduced at very low copper concentration (ZC3 sample) doped in ZnO. Since, the ionic radii of Cu^2+^ (0.057 nm) and Cu^1+^ (0.06 nm) are very similar to Zn^2+^ (0.06 nm) ion and have stable electronic configuration with Cu^2+^ (3d^9^) and Cu^1+^ (3d^10^) having low formation energy, it is favorable that Cu cations substitutes the Zn cations in the unit cell of ZnO. The interaction between acceptor defects and intrinsic donor in ZnO may occur by the capture of electron from the lattice. The conductivity of lowest Cu content (ZC3) decreased by one order of magnitude in comparison to pure ZnO thin films. The acceptor like behavior of Cu defects, which can reduce the concentration of conducting electrons provided by the shallow defect donors, might be responsible for the reduction in electrical conductivity of ZnO in ZC3 sample. It suggests that Cu substitution in ZnO results in Cu^2+^ state or/and in Cu^+1^ state at this concentration. When, Cu doping is increased (ZC5 sample), the conductivity, carrier concentration and mobility are also increased. These results can be explained as the addition of more Cu in ZnO film exceeds the solubility limit of Cu in ZnO, a metallic state (Cu^0^) is formed, which in turn introduces additional free carriers (electrons). Thus, increase in carrier concentration due to the presence of metallic copper in ZnO leads to increase in conductivity and mobility. However, further excess doping of Cu (ZC7 sample) results in decrease in mobility of charge carrier. Whereas, there is increase in carrier concentration due to the presence of metallic copper in ZnO resulting in increase in conductivity. The decrement in mobility may be attributed to the (1) segregated Cu around grain boundary combined to form Cu nano particle which discontinue the conducting path of charge carrier, (2) crystallinity is deteriorate leading to the smaller grain size which increases the grain boundary scattering.

There are various reports which suggest that Cu ions serve as an active luminescent centre in the host material and its emission properties are strongly dependent on the doping concentration and defects in the host matrix^45,46^. Cu in ZnO exhibits range of emission lines from the ultraviolet to the infrared region, which are well reported in the powder or the thin film form of ZnO^15,47,48^. Room temperature photoluminescence (PL) spectra of pure ZnO and ZnO:Cu samples of different Cu concentration are shown in Fig. 4(b). It is interesting to note that the PL spectra of ZnO and ZnO:Cu thin films show a broad green band, whose intensity is maximum in sample with lowest Cu concentration and further increase in Cu concentration results in decrease in intensity of green band. In the case of the undoped ZnO, the contribution of oxygen vacancies plays a significant role in the origin of green peak at ~520 nm. However, the exact mechanism of green emission in ZnO is not yet clear and energy levels of various defects reported in literature differ greatly. Moreover, Fig. 4(c) display various energy levels within forbidden band of ZnO:Cu for better visualization of the green emission due to Cu impurity in ZnO. Several reports suggest that the oxygen vacancies and Cu impurity are responsible for green emission in ZnO. Dingle suggested that the charge transfer from impurity states (Cu^2+^) to perturbed valence band is most likely cause of green luminescence in copper doped ZnO^15^. The extension of this model as suggested by Garces *et al*.^16^ predicted that Cu may exist either in Cu^+^ or Cu^2+^ state. It is reported that monovalent state (Cu^+^) will give structure less emission due to donor–acceptor pair recombination in which transition from the copper acceptor to shallow donor impurity occurs. In present investigations, green luminescence in pure-ZnO, may be due to the presence of native defects in ZnO. Moreover, there is little possibility of oxygen vacancy, which is also a cause of green emission in ZnO. However, a clear enhancement in green emission in ZnO:Cu samples indicates the role of Cu in green emission. It also suggests that the enhanced green emission is predominantly due to copper-impurities. At the lowest Cu concentration in ZnO, Cu takes the Zn site and forms the divalent copper ion Cu^2+^, which easily captures the electron to become Cu^+^ with full filled or half filled outer electronic structure. Thus, it forms accepter level below the conduction band, which is responsible for the enhancement of green PL. Furthermore, the intensity of green luminescence peak decreases with increasing copper concentration, which can be explained on the basis of solubility limit of Cu in ZnO. Beyond the solubility limit, the Cu precipitates to form Cu nanoparticles and Cu^2+^ and Cu^1+^ ions deplete in the matrix resulting in decrease of green luminescence. When, Cu concentration is not too high (less than 3 at. %), one can suggest that Cu atoms substitutes the Zn ions, and then the Cu atoms donate two electrons in the process of band formation and charge can transfer from Cu^2+^ to valance band resulting into green emission. This is the main reason behind the rapid increase in luminescence intensity with increasing Cu^2+^ ions. Whereas, for higher Cu concentration, excess Cu atoms can be energetically favorable to agglomerate into metallic copper clusters or CuO, but in the present case, CuO phase could not be detected within instrumentation limit thus it can be concluded that for higher Cu concentration, only Cu nano particles are formed. Hence charge transfer from Cu^2+^ to valance band is quenched, which leads to the decrease in green luminescence peak. Thus, decrease in n-type conductivity and appearance of green emission after copper doping in ZnO confirm that the Cu is doped in ZnO lattice and it is the cause of green emission.

**Figure 5:**
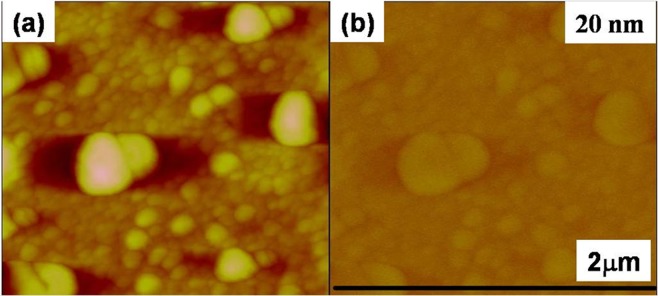
(**a**) Topographic AFM and (**b**) MFM image at 20 nm lift height of pure ZnO thin film shows no phase contrast revealing non magnetic nature.

The enhanced RTFM is observed by viewing the magnetic domain in room-temperature magnetic force microscopy (MFM) measurements. For all the films, the topographical and the corresponding MFM images were taken with an image size of 5 μm × 5 μm with a different lift distances in a phase detection mode during MFM measurements. The MFM magnetization map is dependent of the AFM topography image of the nanocomposite surface films. The images of topography (left) and magnetic force gradients (right) of ZnO are shown in Fig. 5.

There is no clear phase contrast in un-doped ZnO at very small lift height of 20 nm as shown in Fig. 5, indicating a very weak magnetic response. At lower lift height there is no change in images AFM and MFM images almost similar with no magnetic signal which shows that undoped ZnO thin film is non magnetic. The most recent results have demonstrated that oxide thin films or nanostructures do not need magnetic cations to become magnetic. RTFM in pure ZnO is also found to increase upon thermal annealing^49^ and explained by the formation of the anionic vacancy clusters. The literature also reports that RTFM is attributed to Zn vacancies^50,51^ oxygen defects^52^ and Zn clusters^29^. Therefore, presence of weak magnetic signal in ZnO film is not surprising. It could stems from variety of defects.

**Figure 6:**
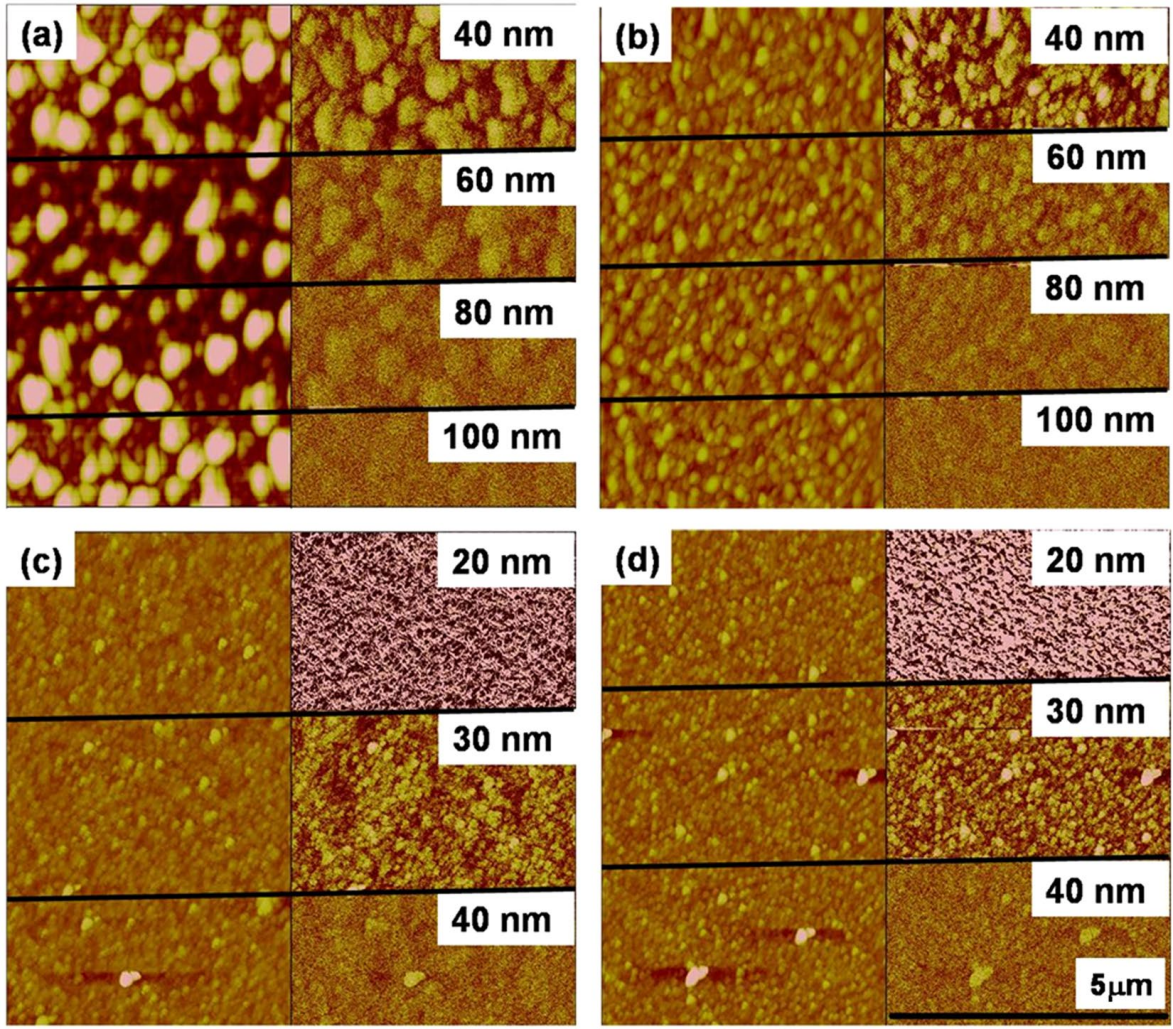
Topographic AFM (left) and MFM image (Right) of ZnO:Cu thin films of (**a**) ZC3, (**b**) ZC5, (**c**) ZC7, (**d**) ZC15. MFM image shows phase contrast indicating the magnetic nature.

On the other hand, under the same scanning conditions, Cu doped ZnO samples show a clear magnetic response as demonstrated in Fig. 6. The magnetic domain structure observed in the ambient condition (300 K) shows granular features for all the Cu doped ZnO thin films. The attractive and repulsive forces are characterized as a dark and bright area in the MFM images respectively. A clear correlation between magnetic and topographic features is observed as bigger topographic grains show higher magnetic forces and smaller topographic features exhibit smaller magnetic domains. It suggests that topographic grains are actually magnetic. As the tip was magnetized in the upward direction, the color of the scale bar represents the strength of the perpendicular component of magnetization to the surface. The strength of the perpendicular component of magnetization is decreased on increasing the distance between tip and sample and magnetic grains disappeared at large distances. It could be seen clearly that the strength of magnetization is larger in ZC3 and ZC5 samples as magnetic domains are still visible at 80 nm lift height (Fig. 6(a,b)). Moreover, on further increasing the Cu concentration, the strength of magnetization is reduced as evidenced from Fig. 6(c,d).

**Figure 7:**
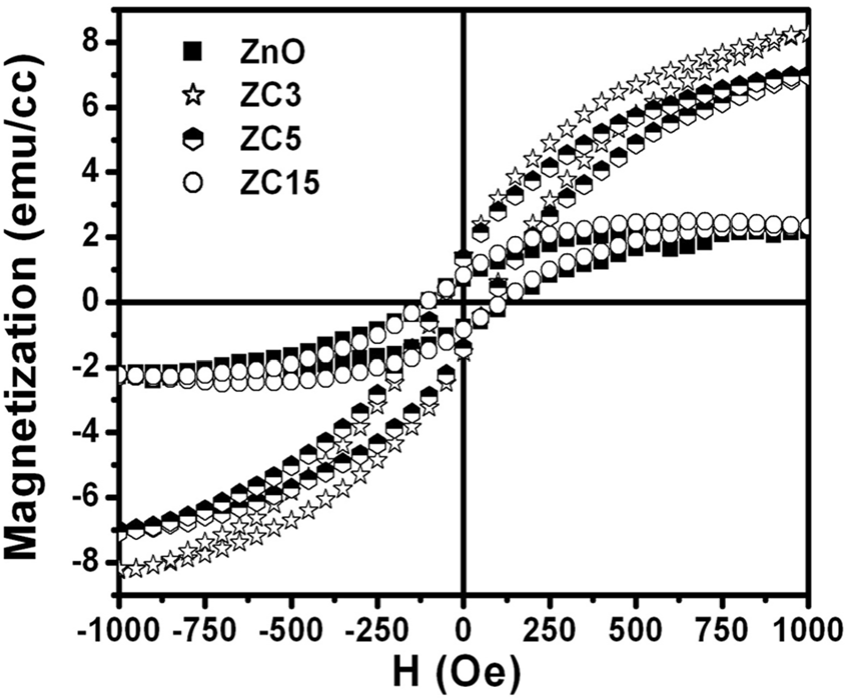
Room temperature M-H curve for ZnO and ZnO: Cu thin films with varying Cu concentration shows hysteresis response confirming the ferromagnetic behavior.

Magnetization studies on the as-deposited and Cu doped ZnO thin films have also been performed using a highly sensitive vibrating sample magnetometer for quantification of magnetic behavior. Similar to MFM results, all the samples show ferromagnetic ordering (FMO) at room temperature. Figure 7 shows the M-H curves for the pure and Cu doped ZnO films recorded at 300 K. The hysteresis response in the M-H curve confirms ferromagnetic behavior of the as-deposited and doped thin films. The saturation magnetization is found to increase from 2.0 emu/cc to 8 emu/cc with varying Cu concentration from 0 to 3 at%, respectively in the ZnO thin films. However, if concentration of the Cu is further increased up to 15 atomic %, the saturation magnetization is decreased and attained at level of the un-doped ZnO thin films. It may be noted that the measured value of the coercivity is not changed, when Cu is doped in the ZnO.

The large enhancement in RTFM in n-type conducting Cu doped ZnO films clearly indicate that the ferromagnetism may not arises only due to oxygen vacancies or carriers mediated. Indirect double exchange model^28^ which was based on first principle calculations proposed that alignment of localized large moments of Cu in the vicinity of Vo are mediated by large sized vacancy orbitals. This model alone could not justify the observed results as pure ZnO thin film having oxygen vacancies showing almost non magnetic or very weak magnetic nature and Cu doped ZnO have very strong ferromagnetic signal. This convinces that only oxygen vacancy are not responsible for ferromagnetic nature and lead to anticipate some other prospects. Moreover, some structural inhomogeneity and formation of bound magnetic polarons due to structural defects are also responsible for observed ferromagnetism. Recently, Pan *et al*., reported that overlapping of bound magnetic polarons (BMPs) is the main ferromagnetic origin of ZnO/Cu and show stronger ferromagnetism for 3at% of Cu^30^. According to them, ferromagnetic coupling of Cu_Zn_ and V_Zn_ form overlapping of BMPs Cu^2+^ − V_Zn_ − Cu^2+^ or Cu^2+^−V^−^_Zn_−Cu^3+^ which are responsible for the long ranged ferromagnetism upto 3at% of Cu and if spin orientation is randomly oriented for isolated Cu^2+^ then there will be no ferromagnetism^30^. These theoretical predictions are very well matched with our experimental findings that shows stronger ferromagnetism 3 at% and starts decreasing for higher Cu. Durst *et al*. also gave a simplest model of formation of bound magnetic polarons due to the defects created by impurity incorporation in the host^31^. These polarons are coupled with 3d moments within their orbits^29^. The ferromagnetic ordering is stabilized due to the spin-spin interactions between magnetic ions, which are induced by magnetic polarons. Unlike the carrier mediated mechanisms, bound magnetic polarons may also be a cause of origin of RTFM in magnetic ion doped semiconductors with low carrier densities. Furthermore, the decreased magnetization at higher Cu content in ZnO films may be either due to the anti ferromagnetic ordering between Cu ions due to shorter distance between them or formation of Cu nanoparticles at higher concentration, which induces alloy disorder in the sample. It is also supported by MFM figure that the magnetic grains are small and very close to each other as compared to pristine and low Cu concentration doped ZnO film.

## Conclusion

In summary, ZnO thin films doped with varying Cu concentration are grown by atom beam sputtering technique. The phase and structural analysis carried out by XRD suggested that incorporation of Cu enhances the crystallinity of the host thin films when it is doped in ZnO up to moderate concentration of about 3 atomic %. Enhancement in green luminescence for lower Cu concentration indicates that Cu substituted on Zn site, whereas, high concentration of Cu suppressed the green emission. Saturation of magnetic moment in ZnO:Cu samples also shows similar trend as observed in case of intensity of green luminescence with Cu concentration. It is interesting to note that both optical and magnetic properties show strong correlation with Cu content. Both are enhanced at low Cu concentration and suppressed at higher Cu content in ZnO. The results are significant for indicating the application of ZnO:Cu film with moderate Cu concentration in fabrication of spin LED.

## Material and Methods

ZnO and ZnO:Cu thin films were deposited on Si and quartz substrate by atom beam co-sputtering technique at Inter-University Accelerator Centre (IUAC), New Delhi. The Cu foils having area of 5 mm × 5 mm were symmetrically glued on 2 inch ZnO target and co-sputtered using neutral Ar atom beam of energy of 1.5 keV at an incidence angle of 45°. The details of synthesis process of pure ZnO films have been reported elsewhere^33,34^. Furthermore, the number of glued Cu foils was varied to have different concentration of Cu. Henceforth, samples named ZnO and ZC3, ZC5, ZC7 and ZC15, are corresponding to ZnO thin films having 0,3,5,7 and 15 atomic % of Cu. Structural properties were studied using grazing angle incidence x-ray diffraction (XRD) technique. The XRD pattern was recorded with Bruker D8 advance diffractometer at grazing incidence angle of 2° using a CuK_α_ source. Band gap and absorption spectroscopy of the films were investigated using a dual beam Hitachi U3300 spectrophotometer. The micro-Raman scattering were carried out using a Ranishaw model Invia Raman Microscope with Ar ion excitation laser operating at a wavelength of 514 nm. Electrical properties were determined by the Hall measurements in a van der Pauw four-point configuration at room temperature with 1 T magnetic field using HMS-3000. The MFM data were recorded using a scanning probe microscope by Nanoscope-III with Co-coated low-moment probe tips. The room temperature magnetization measurements of Cu-doped ZnO thin film are done using Micro-sense vibrating sample magnetometer (VSM) setup having sensitivity of about 0.1μemu at the Central Electronics Engineering Research Institute (CEERI), Pilani.
